# Identification of Mode Shapes of a Composite Cylinder Using Convolutional Neural Networks

**DOI:** 10.3390/ma14112801

**Published:** 2021-05-25

**Authors:** Bartosz Miller, Leonard Ziemiański

**Affiliations:** Faculty of Civil and Environmental Engineering and Architecture, Rzeszów University of Technology, al. Powstańców Warszawy 12, 35-959 Rzeszów, Poland; ziele@prz.edu.pl

**Keywords:** shell, layered composites, mode shapes, identification, machine learning

## Abstract

The aim of the following paper is to discuss a newly developed approach for the identification of vibration mode shapes of multilayer composite structures. To overcome the limitations of the approaches based on image analysis (two-dimensional structures, high spatial resolution of mode shapes description), convolutional neural networks (CNNs) are applied to create a three-dimensional mode shapes identification algorithm with a significantly reduced number of mode shape vector coordinates. The CNN-based procedure is accurate, effective, and robust to noisy input data. The appearance of local damage is not an obstacle. The change of the material and the occurrence of local material degradation do not affect the accuracy of the method. Moreover, the application of the proposed identification method allows identifying the material degradation occurrence.

## 1. Introduction

The design of composite structures requires the selection of appropriate values of certain control parameters that describe both the structure itself and the material that it is made from [1,2]. The number of composite layers and their stacking sequence are, among others, frequently used for tuning selected properties of composite structures [3] (e.g., desired vibration frequency spectrum [4,5], buckling behavior [6], or structure’s stiffness [7]). The values of the parameters that give the expected results are often determined through the optimization process [8], namely through repeated calculation of the so-called objective function, which is minimized in the space of varying parameters (e.g., lamination angles).

**Figure 1:**
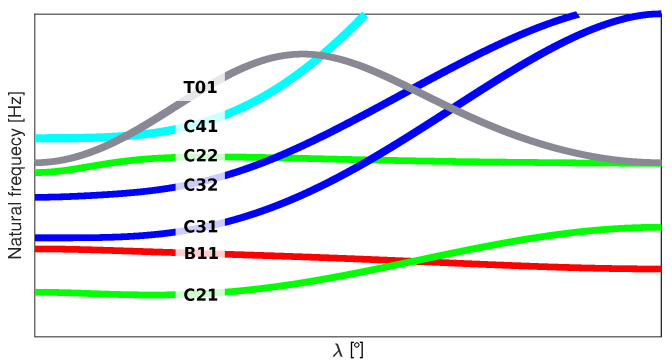
An example of natural frequencies crossing; three-layer composite cylinder with stacking sequence [λ,0,λ].

As shown by Ruiz et al. in [9] and Bunting et al. in [10], in the process of optimization of dynamics features, some problems with the nondifferentiable objective function may arise. They are often caused by swapping the vibration mode shapes for different values of control parameters governing the optimization process (see Figure 1) or the occurrence of double (repeated) natural frequencies and corresponding mode shapes. As a result, ambiguity in the solution of the optimization problem may be encountered.

Figure 1 explains this phenomenon, where the sequence of natural frequencies corresponding to particular mode shapes is amended due to changes in the structure’s parameters and the discontinuity of the derivative of the objective function appears. The solution to these problems is known as natural frequency tracking in the domain of the optimization parameters; it eliminates the problems of nondifferentiability of the objective function. Natural frequency tracking requires permanent identification of the mode shapes corresponding to natural frequencies for each point in the control parameter space of the objective function.

In dynamic analysis, the objective function requires an eigenproblem solution giving natural frequencies and corresponding mode shapes without identifying the type (class) of the mode shape. This makes it impossible to track natural frequencies and to capture the phenomenon of modes crossing, i.e., the changes in the sequence of natural frequencies corresponding to particular mode shapes. The application of mode shape identification, that is, the designation of the natural frequency and the corresponding vibration mode shape with a suitable “mode shape identifier”, is important not only in first-order (gradient-based) optimization. It also improves the convergence of the solution in zero-order optimization (using, e.g., evolutionary algorithms) and gives more accurate results [6,11]. In [6,11], Miller and Ziemiański applied a mode shape identification procedure based on analytical “search” of mode shape nodes and antinodes along with the axial and circumferential directions of a composite cylinder. The procedure is hereafter called the “analytic” identification procedure and is sensitive to inaccuracies, errors, noise, and changes of parameters. In the analyzed task, it proved its effectiveness only within a limited range of variation of parameters describing the investigated model.

The mode shapes identification is also advisable in the process of updating computational models [12,13] and in structural health monitoring (SHM) [14]; therefore, many researchers have been working on this problem using different approaches: Zernike moment (ZM) descriptors [12], radial Tchebichef moment (RTM) descriptors [13], and Fourier descriptors [15]. The geometrical descriptors for mode shapes identification use the concept of image analysis, introduced in [16] and based on the theory of two-dimensional moment invariants for planar geometric figures. ZM descriptors are advisable in the analysis of circular and spherical images, but in the case of structures of other shapes (e.g., rectangular), a coordinate transformation (mapping into a unit circle) is necessary. The RTM descriptor, as stated in [13], is superior to the ZM descriptor in terms of mode shapes identification but is still intended to analyze circular images. Fourier descriptor does not only aim at analyzing circular images but still operates on plain areas. The aforementioned descriptors also have the mode shape to be acquired using fine spatial resolution. To overcome this difficulty, other image processing approaches [17] supported by machine learning [18] and using the geometry description capabilities known as NURBS (nonuniform rational B-spline) [19] are used. While the NURBS-based approach operates directly on curves and areas and can be applied to describe also 3D geometry, mapping to a unit primitive and fine spatial resolution is still necessary.

La et al. applied principal component analysis and support vector machines in the identification of mode shapes [20]. However, he only considered only flat structures; the obtained accuracy of two-dimensional mode shape identification was at the level of approximately 98%.

In the case of three-dimensional structures, the above-mentioned methods require an advanced preprocessing of mode shapes to map them to plain areas. Moreover, the investigation of displacements in directions other than perpendicular to the plain area the investigated region is mapped to can prove problematic since only out-of-plain displacements can be captured through these approaches. Another important feature of these methods is their need for an accurate representation of the mode shape, which makes them sensitive to discretization errors [13].

Recently, there has been tremendous growth in the applications of Convolutional Neural Networks (CNNs) in engineering, particularly in areas such as computer vision and pattern recognition, object detection, speech recognition, biomedical systems, and natural language processing. This was possible through the use of new learning algorithms based on the deep learning technique [21,22,23]. Unlike classical neural networks (currently often referred to as shallow networks), a CNN is built from neurons ordered in three directions: width, height, and depth, which allows for feature detection in the image as well as in time series [24,25]. CNN processing capabilities have been used repeatedly in applications related to computational mechanics [26], vibration analysis [27], and SHM [28,29,30]. In [31], a new network (named TICNN) was proposed for feature extraction and classification of models with external disturbances.

In the following paper, a successfully developed new approach for the identification of vibration mode shapes of multilayer composite structures is discussed. To overcome the limitations of the above-described approaches (2D analysis, high spatial resolution of mode shapes description), CNNs are applied to create a three-dimensional mode shapes identification algorithm with a significantly reduced number of mode shape vector coordinates. The CNN-based procedure is accurate, effective, and robust to noisy input data and the appearance of local damage is not an obstacle either. The change of the material or the occurrence of local material degradation do not affect the accuracy of the method. Moreover, the application of the proposed identification method allows identifying the material degradation occurrence.

The proposed approach is based on the vibration shapes that can be obtained from experimental measurements. Currently, equipment and modern, efficient computational algorithms (such as digital image correlation (DIC) [32,33] and scanning laser Doppler vibrometry [34]) allow the measurement and the analysis of mode shapes as full-field data, not limited to a few, selected degrees of freedom.

The paper is organized as follows: Section 2 contains the formulation of the problem. Section 3, the main section of the article, discusses the CNN-based identification of mode shapes. Section 4 presents the identification of the occurrence of material degradation. Section 5 contains the discussion of the results. The conclusions and future research directions are reported in Section 6.

## 2. Formulation of the Problem

### 2.1. Solution of the Vibration Problem

The dynamic behavior of a structure (or, more precisely, of its numerical model) is governed by the generalized equation of motion [35]:

(1) $$M\ddot{x}+C\dot{x}+Kx=P,$$

where M is a mass matrix, C is a damping matrix, K is a stiffness matrix, x is a vector of nodal displacements, and $\dot{x}$ and $\ddot{x}$ are the first and the second derivatives of x with respect to time *t*, respectively; P is an external force vector.

Equation (1), for the free vibration analysis, is simplified to:

(2) $$M\ddot{x}+Kx=0,$$

where P and C are neglected (i.e., excitation does not occur and the damping is ignored). This leads to the generalized eigenproblem [36]:

(3) $$K\Phi =M\Phi \Omega^{2},$$

where Φ matrix is built from the eigenvectors $\phi_{i}$ describing the mode shapes corresponding to angular frequencies contained in the diagonal matrix Ω. Each of the angular frequencies after dividing by 2π gives an ordinary frequency:

(4) $$f_{i}=\frac{\omega_{i}}{2\pi },$$

called the natural frequency $f_{i}$ here.

Each of the $\phi_{i}$ mode shapes (also called natural vibration shapes or resonant shapes) describes the maximal deformation of the structure when it vibrates harmonically with a corresponding $f_{i}$ natural (resonant) frequency. The set of mode shapes is treated here as a main source of information on the investigated structure.

### 2.2. Investigated Structure and Its Finite Element Model

The structure under study is a cylinder with radius R=0.6103 m and length l=6.0 m. The shell of the cylinder is a multilayer composite; each of the *n* layers of composite material has the same thickness with the total thickness of the shell—regardless of the number of layers of the composite—being constant at t=0.016 m. The angles of the reinforcement fibers can be different for each layer. The composite material constants, based on [37], correspond to graphite-epoxy composite material: $E_{1}=141.9$ GPa, $E_{2}=9.78$ GPa, $\nu_{12}=0.42$, $G_{12}=6.13$ GPa, and ρ=1445 kg/m${}^{3}$.

**Figure 2:**
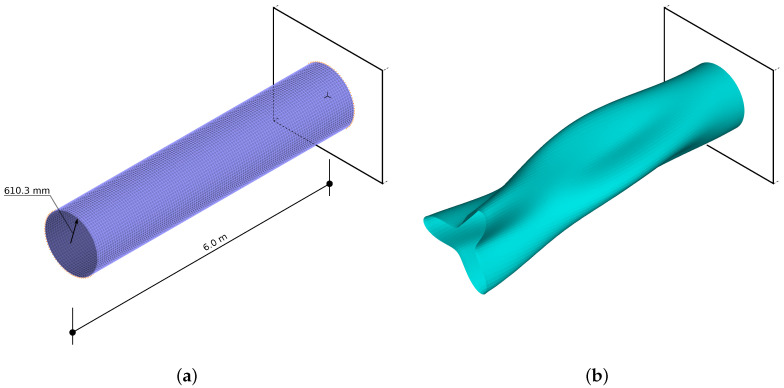
The finite element (FE) model (**a**) and an example of C32 mode shape (**b**).

A regular finite element (FE) mesh (see Figure 2) was used in the FE model, with the number of FEs along the cylinder axis equal to 80 and circumferentially equal to 120. In total, this leads to a model with 9680 nodes and 58,000 degrees of freedom. A four-node multilayer shell FE (first-order shear theory) was applied; in the Adina FE code ([38], used in all FE calculations) it is called MITC4. The FEM model was built using the experience gained by the authors in previous research (see [6,11,39,40]), where FE convergence was verified and the results from different FEM systems were compared.

The cylinder is built in at one end by blocking the displacements of all nodes located at the selected end of the cylinder.

### 2.3. Convolutional Neural Networks

The convolutional neural network is a specialized kind of neural network designed for advanced data processing, with a gridlike topology [41]. Examples of the data that CNN was created to analyze include, e.g., images (treated as a two-dimensional grid of pixels), time series data [27] (one-dimensional grid of data sampled from an observed signal at regular time intervals), and multidimensional data. Convolutional networks are used with great success in numerous practical applications and have thus become the standard for recognition systems and image or video processing [27]. In recent years, CNNs have also been readily used in SHM systems, mainly for vibration analysis [14,28,29,30].

The main difference between a classical neural network (now called a shallow neural network) and a convolutional network is the fact that a shallow network uses—as the main operator—general multiplication, whereas a CNN uses convolution (i.e., an operation on two functions that produces a third function). In convolutional network terminology, arguments to the convolution are often referred to as the input and the kernel whereas the output is referred to as the feature map.

CNN can be treated as a grid made up of segments (layers), such as: the convolution layers (for feature extraction), pooling layers (dimension reduction), batch normalization layers (enabling independent learning of selected network parts), and activation layers (here, rectified linear units, ReLU).

**Figure 3:**
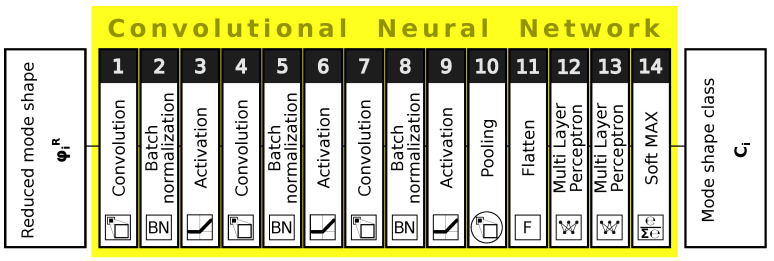
An example of convolutional neural network (CNN) architecture.

In the paper, CNN is used as a classifier applied to identify a “class” of vibration mode shape of a composite cylinder using a three-dimensional matrix of nodal displacements as a source of input data. CNNs are trained using the RMSProp algorithm [41]. The architecture of CNNs applied here is summarized in Table 1 and shown in Figure 3. Neural network’s hyperparameters are determined by trial and error procedure.

All simulations using CNNs were performed in the commercial code Mathematica (V12.0, Wolfram Research Inc., Champaign, IL, USA) environment [42].

## 3. Mode Shapes Identification

### 3.1. The Analytical Approach

The identification of mode shapes significantly improves the results of the optimization of stacking sequence in composite structures (see [6,11]); therefore, it is analyzed in detail in this paper.

The initial FE model is biaxially symmetric. The mode shapes of natural vibrations can be divided into Axial (A0*m*, m=1,2,3,…), Torsional (T0*m*, m=1,2,3,…), Bending (B1*m*, m=1,2,3,…), and Circumferential ones (Cwm, w=2,3,4,…, m=1,2,3,…).

The mode shape code, Xwm, shows that the mode shape family (X is replaced by A for axial, T for torsional, B for bending, and C for circumferential mode shape), circumferential wave *w*, and axial mode *x*. As a rule, different mode shapes correspond to unique natural frequencies; for axisymmetric FE models, close values of the natural frequencies of adjacent vibration modes can be observed (e.g., $f_{1}\approx f_{2}$).

Mode shapes identification and assigning them to appropriate classes simplifies the analysis and allows building simple and effective tools for the optimization and diagnosis of the structure under study [6,11]. For the initial model (see Figure 2), with no defects and/or material degradation, it is possible to create an analytical procedure for the identification of mode shapes (see [11]). The procedure in the form described in [11] relies on the analysis of the movement of the FE model node with the highest displacement magnitude. This approach, which is fast and effective for biaxially symmetric structure, is not reliable enough for the structure with local material degradation and/or geometric uncertainties, which in turn leads to the loss of symmetry (especially for large areas of material degradation). In what follows an automatic, neural network-based, identification of mode shapes is presented. However, the analytical method is still applied in the new approach; the learning and testing patterns were built using the results of the identification performed by the analytical procedure.

### 3.2. Neural Network Based Mode Shapes Identification

The range of the analyzed natural frequencies was limited here with the value 100 Hz, which seems to be a reasonable restriction from the point of view of the civil engineering analysis. The mode shapes corresponding to the natural frequencies lower than 100 Hz are the following ones: A01 (one axial mode), B11 and B12 (two bending modes), C21, C22, C23, C31, C32, C33, C41 and C42 (eight circumferential modes), and T01 (one torsional mode). The majority of them are—for biaxially symmetric structures—double mode shapes (i.e., two corresponding natural frequencies are almost equal and the corresponding mode shapes differ only in rotation about the structure axis of symmetry), and the overall number of the analyzed frequencies thus reaches 22 (10 of 12 considered modes are double ones).

**Figure 4:**
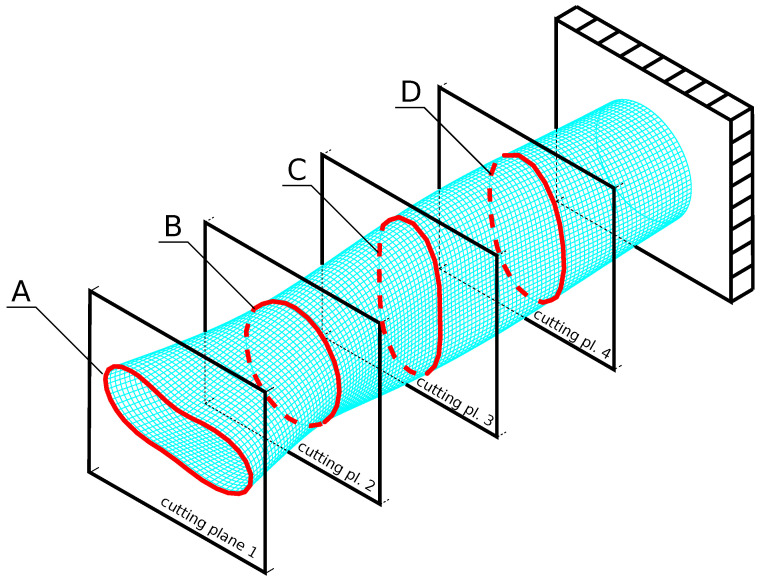
Selection of four FE model cross-sections for mode shape description.

To identify the above-listed mode, CNNs are applied. Although CNNs are particularly suited to image analysis, they can also efficiently analyze numerical sets. The training of CNN is performed using a set of examples (called patterns); here, each of the examples (patterns) consists of a 240-element input matrix (3×20×4) describing the analyzed mode shape (three mode shape components—displacements along three Cartesian coordinate system axes—in 20 nodes of four cross-sections of the FE model (see Figure 4)), and one-element desired output showing the mode shape name (obtained from the analytical identification procedure). The CNN output may be presented using either the usual classification approach (the name of the identified mode shape) or a vector, where each of the vector elements shows the level of similarity to one of the considered mode shapes. The output vector contains the values from the range (0,1), and the identified mode shape is chosen as the one corresponding to the maximal element of the output vector.

To reduce the dimensions of the input matrix, the description of each mode shape is reduced, as mentioned above, to 3×20×4 matrix. The selected 20 nodes in each of the selected four cross-sections are chosen as every fourth node in each of the cross-sections A, B, C, and D in Figure 4; the cross-sections are located 6.0 m, 4.5 m, 3.0 m or 1.5 m away from the fixed end of the cylinder.

To verify the accuracy of the proposed identification method, four different numbers of layers of composite material were considered: n=4,8,16,32. For each value of *n* (with the shell thickness constant for all the considered cases), 2000 random lamination angles sets were generated, and in each case, 12 mode shapes were considered (as described above: one axial mode, two bending modes, eight circumferential modes, and one torsional mode). Only axial and torsional modes are single modes; however, in the case of double modes, the corresponding mode shapes are not identical; they are rotated around the axis of the cylinder. The inclusion of both double mode shapes increases the accuracy of the procedure. The overall number of considered modes reached 24 (2×12) for each of the models since axial and torsional modes (single ones) were also repeated. Altogether, 192,000 patterns were obtained (one model generated 24 patterns; the number of models was equal to 2000 for each number of *n*).

**Figure 5:**
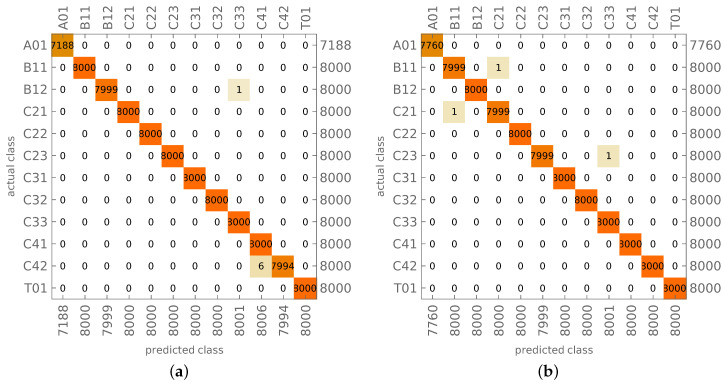
The results of CNN-O mode shapes identification: (**a**) learning, (**b**) testing.

The patterns were divided into learning and testing sets: all the cases generated for n=4 and n=16 composite layers were used as learning patterns while the cases with n=8 and n=32 layers were used at the testing stage. The number of learning patterns was the same as the number of testing patterns—in both cases, 96,000. However, the analytical identification procedure—used as the reference procedure—failed to identify A01 mode shape in some of the considered cases, and the number of corresponding patterns was slightly smaller (see Figure 5).

The results of learning and testing of CNN network (called in what follows CNN-O) are shown in Figure 5. The figure shows the confusion matrix, where the vertical axis presents the target mode shape (identified using the analytical reference procedure) and the horizontal axis shows the CNN-identified (predicted) mode shape. The numbers on the right-hand side or below the plot show the overall number of cases in a particular row (the proper number of cases representing a particular mode shape) or column (the number of cases predicted as a particular mode shape).

In case of faultless identification of all mode shapes, there should be no cases outside the diagonal of the confusion matrix and the number of cases in the corresponding row and column should be equal. There are a few errors in the results shown in Figure 5; however, their number is negligible. The level of accuracy of the identification of testing mode shapes reaches almost 100%.

It has to be noted that in some selected cases, a result classified as incorrect in comparison with the reference analytical procedure may be in fact correct, because the analytical procedure also may incorrectly identify the mode shape in question. However, such cases are few and do not affect the accuracy of the CNN procedure.

**Figure 6:**
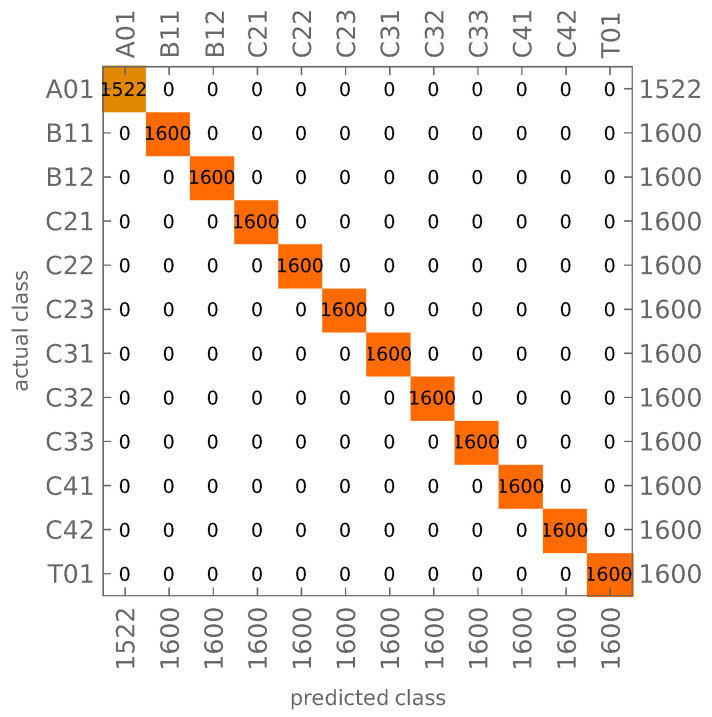
The results of CNN-O mode shapes identification for different material.

The trained CNN-O was also verified using mode shapes obtained from the same FE model with some material constants altered; the values of Young’s moduli were changed to $E_{1}=113.52$ GPa and $E_{2}=10.73$ GPa (while the original moduli were $E_{1}=141.9$ GPa, $E_{2}=9.78$ GPa). The results of mode shapes identification are shown in Figure 6; the number of models with altered material constants equals 800 (200 different lamination angles cases for each *n*). The results shown in Figure 6 prove that the CNN trained on a model with constant shell thickness and material properties is able to properly predict mode shapes for a structure made of a different material.

Further verification of the robustness of the proposed mode shapes identification procedure involves artificial, random disturbing of mode shapes obtained from numerical simulations. Random noise can emulate some measurements errors, inevitable during real experiments. The mode shapes obtained from numerical simulations were processed in three consecutive steps:

1. each of the four cross-sections was shifted by a random vector (the same for the whole cross-section),
2. each node on each of the four cross-sections was shifted by a random vector (unique for each node),
3. the accuracy of each mode shape element was truncated to *l* significant digits.

In each case, the considered random noise was of a Gaussian distribution. The above described three steps can be described using simple formulas. The random shift of each mode shape cross-section is governed by the Equation:

(5) $$P^{1}=P^{0}+G^{s}\left(0,\sigma_{1}\right)$$

where $P^{0}$ is the original location of the considered cross-section (i.e., $P^{0}$ contains in-plane coordinates of each of the 20 points of a particular cross-section), and $G^{s}\left(0,\sigma_{1}\right)$ is a random shift vector (its coordinated are generated using Gaussian distribution with mean equal to 0 and $\sigma_{1}$ standard deviation),

(6) $$P^{2}=P^{1}\times G^{n}\left(1,\sigma_{2}\right)$$

where $P^{1}$ are the randomly shifted (see Equation (5)) coordinates of each of mode shape nodes and $G^{n}\left(0,\sigma_{2}\right)$ is the random coordinate multiplayer, unique for each mode-shape node (Gaussian distribution with mean equal to 1 and $\sigma_{2}$ standard deviation),

(7) $$P^{3}=N\left(P^{2},l\right)$$

where N(·,l) is an operator of truncation to *l* significant digits.

**Figure 7:**
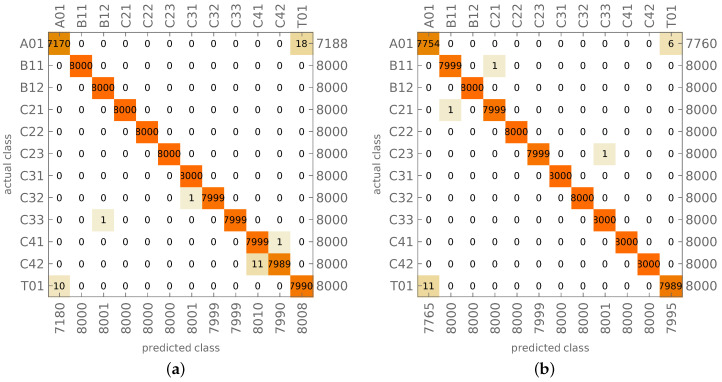
The results of CNN-N mode shapes identification using noisy data ($\sigma_{1}=0.0005$ and $\sigma_{2}=0.25$): (**a**) learning, (**b**) testing.

The network trained and tested on noisy data is called in what follows CNN-N. Different values of $\sigma_{1}$, $\sigma_{2}$ and *l* were considered; for each of them, the proposed identification method is robust and guarantees a high identification accuracy. Figure 7 shows the identification results obtained for $\sigma_{1}=0.0005$ and $\sigma_{2}=0.25$. The value of *l*, while it is not lower than 4, has negligible influence on the results.

In Table 2, we present the number of wrong identification cases obtained during learning and testing for all of the above-described cases. As can be seen, the percentage of errors is small (the values in the parenthesis show the overall number of mode shapes being identified in a particular case).

It should be noted that the analytical reference procedure properly identifies the mode shape family (axial, bending, circumferential, and torsional) only for noised mode shapes with standard deviations not higher than $\sigma_{1}=0.00005$ and $\sigma_{2}=0.005$ ($\sigma_{2}$ ten times smaller than the smallest one considered for CNN identification procedure), but the circumferential waves are already misidentified. For $\sigma_{1}=0.00005$ and $\sigma_{2}=0.05$ (the smallest level of noise considered for CNN identification), the analytical identification is wrong in all cases considered, including the misidentification of the mode shape family. Although the analytical procedure can be improved and better results can certainly be obtained, the experience gained by the authors shows that the results will not be comparable to the CNN results. The analytical procedure is not robust to a larger error, even in just one coordinate among the considered three.

In addition to CNNs, the usefulness of deep learning feedforward neural networks (FFNNs) was also verified. The results are gathered in Table 3, and although the mode shapes identification is rather precise, the advantage of CNNs over FFNNs is clearly visible. The reason for the higher accuracy of CNNs may be related to a different processing method. Moreover, as it is shown in Table 2 and Table 3, the number of patterns in learning sets reaches 96,000; CNNs are well suited to analyze such multielement learning sets.

### 3.3. Identification of Mode Shapes Obtained from the Model with Material Degradation

**Figure 8:**
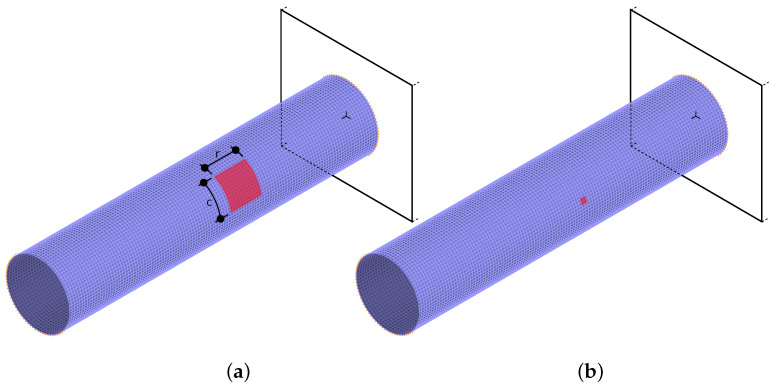
The highest (**a**) and smallest (**b**) areas with a degradation of material model parameters in half of the composite layers.

To verify the ability of the proposed CNN-O network to identify mode shapes obtained from a model with some changes and damages, possibly causing mode shapes changes and/or the lack of model axial symmetry, some local changes were introduced to the model. In a randomly selected area of the model, the material constants of half of the shell layers were reduced, namely, Young’s moduli are in these layers as follows: $E_{1}=14.18$ GPa, $E_{2}=0.978$ GPa (instead of original values $E_{1}=141.8$ GPa, $E_{2}=9.78$ GPa). The area with material constants degradation consisted of *r* rows and *c* columns of finite elements; in all the cases, r=c, so the degradation area was—for simplicity—considered to be “square” (in fact, it is a section of the cylindrical shell). The location of this square area was selected at random without any limitations over the entire shell; the size was limited up to 144 elements (r×s, for r=s≤12) so its biggest size was as high as approximately 1.5% of the whole area of the shell (see Figure 8) while the smallest size (2×2 elements) was as small as 0.04% of the whole area of the shell.

Accordingly, all the cases with n=4 and n=16 composite layers were used as learning patterns, whereas the cases with n=8 and n=32 layers were used at the testing stage. The number of learning patterns was the same as the number of testing patterns; in both cases, it was equal to 144,000 (96,000 patterns obtained from the models without damage and 48,000 patterns obtained from the models with material degradation). The patterns were FEM-generated for the model with random lamination angles and material degradation zone location and size following the previously described assumptions.

**Figure 9:**
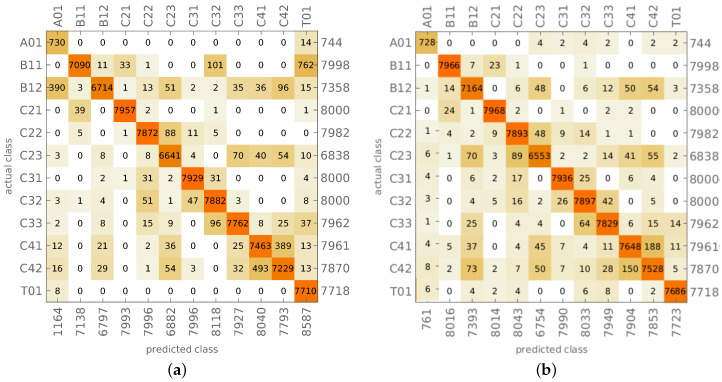
Identification of mode shapes obtained from models with material degradation using: (**a**) CNN-O learned on mode shapes obtained from models without material degradation (see Figure 5), (**b**) CNN-D learned using patterns with and without material degradation.

Figure 9a shows the results of mode shapes identification performed using CNN-O described in the previous subsection (learned using only data from models without material degradation). The overall accuracy of mode shapes identification is equal to 96.01%; the CNN-O network is rather precise also for the models with material degradation. The mistakes are mainly related to the identification of bending shapes (B11, B12) and circumferential shapes with fourth circumferential wave (C41, C42); the reason for these errors may be the limited number of considered cross-sections (only four). The number of examples in each of the identified classes differs from 8000 because the reference analytical procedure fails to identify some mode shapes obtained from models with material degradation.

To improve the results for models with material degradation, the identification CNNs were created again using a few different approaches. The task of the CNN was to identify:

- 24 classes: both the mode shape and the state of the structure (with or without material degradation); the number of mode shapes classes being identified was equal to 24, the additional ones were A01*d*, B11*d*, B12*d*, C21*d*, C22*d*, C23*d*, C31*d*, C32*d*, C33*d*, C41*d*, C42*d* and T01*d* (where *d* stands for material *d*egradation),
- 25 classes: both the mode shape and the state of the structure (with or without material degradation) with an additional 25th class for unrecognized mode shapes; the additional class corresponds to cases where the analytical procedure failed to recognize the mode shape,
- 25 classes (two stage CNN learning): stage I: learning on patterns without material degradation, stage II: additional learning on patterns with material degradation; such approach is suggested for this kind of networks [41], the obtained network is called CNN-D in what follows.

The results are gathered in Table 4; the relative accuracy is related to 24 classes (the classes (·) and (·)*d*, e.g., C21 and C21*d*, were treated as separate ones).

**Figure 10:**
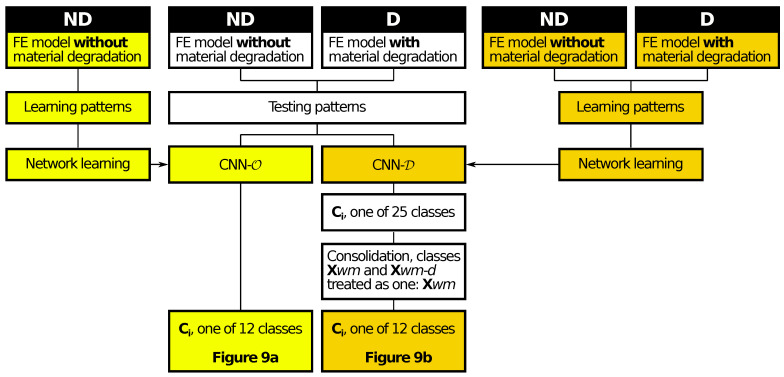
An algorithm applied to identify mode shapes (information about material model degradation neglected); for the results, see Figure 9.

In order to compare the results with the ones presented in Figure 9a, the results obtained from the 25-classes approach were transformed into 12 classes, where the information about material model degradation was neglected (the classes (·) and (·)*d*, e.g., C21 and C21*d*, were treated as one), see Figure 9b and Figure 10 (an algorithm). The accuracy of the mode shape identification, neglecting the information concerning the model state, reaches 98.11% (this value is higher than the accuracy presented in Table 4 since it ignores the differences between (·) and (·)*d* classes).

## 4. Identification of Models with Material Degradation

**Figure 11:**
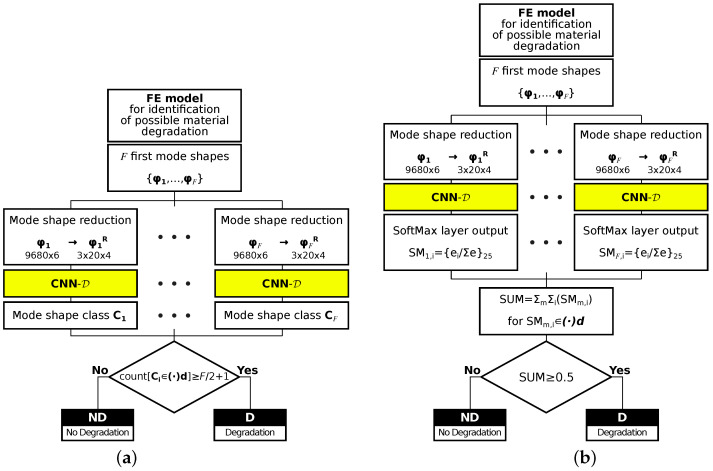
Identification of models with material degradation: (**a**) counting approach (CA), (**b**) probability approach (PA).

The identification procedure described in the previous section was also used to identify the state of the entire model, namely to determine whether material degradation (see Figure 8) took place or not. According to the assumptions described in previous sections, the mode shapes are identified in two states of the entire model ((·) and (·)*d*). The previously created CNN-D (see above) thus produces the output analyzed in one of two ways:

- For each of 24 classes (12 mode shapes with (·) and (·)*d* states), with the 25th class ignored, for each model, 22 first mode shapes are calculated and cases are counted when the identified mode shape belongs to a group of degradated modes ((·)*d* state), when the number of identified degradated modes is equal or higher than 12 the whole model is considered as a model with material degradation; this approach is called in what follows the counting approach (CA); see Figure 11a;
- As it was mentioned earlier, apart from the name of the identified mode shape, CNNs can also return a more elaborate response in the form of a vector composed of probabilities that the analyzed mode shape belongs to considered classes; the probabilities corresponding to degradated modes are summed and divided by the sum of all probabilities when the obtained value is higher than 0.5. The whole model is considered as a model with material degradation; this approach is called in what follows the probability approach (PA) (see Figure 11b).

**Figure 12:**
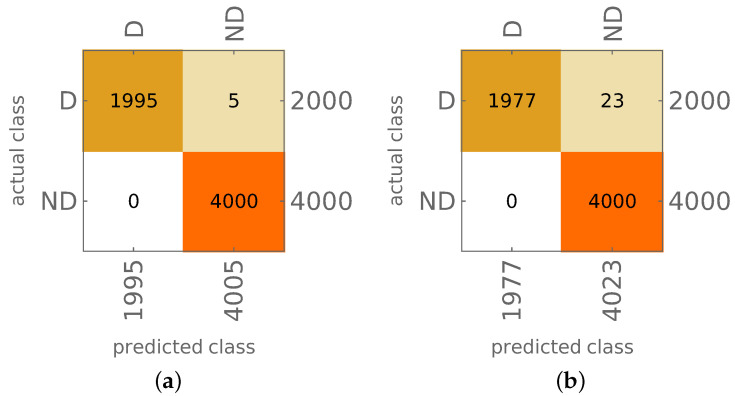
The results of CA model identification: (**a**) patterns used for CNN learning, 4 and 16 composite layers, (**b**) testing, 8 and 32 composite layers.

**Figure 13:**
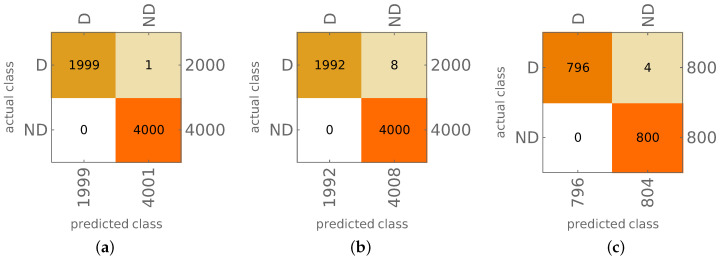
The results of PA model identification (22 mode shapes involved): (**a**) patterns used for CNN learning, 4 and 16 composite layers, (**b**) testing, 8 and 32 composite layers, (**c**) testing of PA procedure for different material.

The results of both approaches are shown in Figure 12 and Figure 13a,b. The appearance of material degradation is indicated with the letter D (as Degradation), the original state is indicated with the letters ND (No material Degradation); the values on the main diagonal show the proper identification of D or ND state of the model (true positives, TP, and true negatives, TN, respectively), whereas the values outside the diagonal show the cases when the ND state is identified as D (false positives) or the D state is identified as ND (false negatives).

The results are precise; in the case of PA, there are no FP results, and the number of FN results equals 9 (0.225% of all the D cases), learning and testing together.

The probability approach was also verified using mode shapes obtained from the same FE model with some material constants altered; specifically, the values of Young’s moduli were changed to $E_{1}=113.52$ GPa and $E_{2}=10.73$ GPa (while the original moduli were $E_{1}=141.9$ GPa, $E_{2}=9.78$ GPa). The results of model degradation identification are shown in Figure 13c.

**Figure 14:**
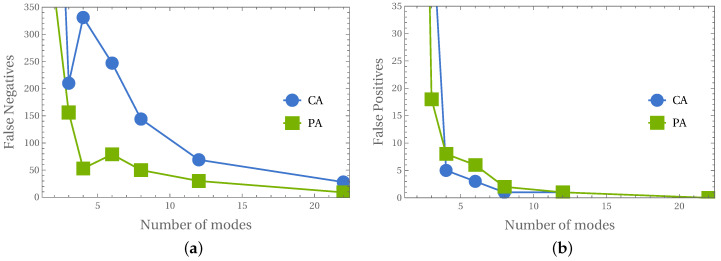
Different number of considered mode shapes in PA and CA: (**a**) false negatives, (**b**) false positives.

Since the obtained results were precise, the analysis of the number of mode shapes involved in the identification procedure was performed. Figure 14 shows the relation of the number of FPs and FNs versus the number of mode shapes involved. The initial sharp drop in the number of FPs and FNs is set almost constant at the level of 10 mode shapes. It can also be observed that the number of FPs is usually about 10 times smaller than the number of FNs.

**Figure 15:**
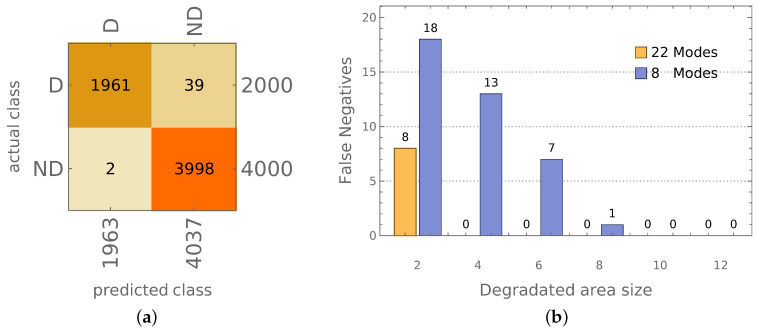
(**a**) The results of PA model identification (testing, 8 mode shapes involved), (**b**) false negatives for different sizes of area with material constants degradation.

The results obtained for only 8 mode shapes involved in the procedure are shown in Figure 15a, while Figure 15b shows the number of FN depending on the size of the area (represented by the number of columns *c* and rows r=c of finite elements) with material constants degradation, with 22 or 8 mode shapes involved in the procedure. The accuracy of the identification of the models with material constants degradation exceeds 95%, even in the case of the smallest area of degradation (only 4 FEs, 0.04% of the whole area of the cylinder) for 8 mode shapes approach and 98% for 22 mode shapes approach. It should be noted here that for all areas apart from the smallest one, no errors of the procedure based on 22 frequencies were observed.

## 5. Discussion of Results

In the statistical analysis of classification problems, whether two-class or multiclass, the most frequently used measures are macroprecision, macrorecall, macro-F1, and accuracy, which are defined in Equations (8) through (11) [29]:

(8) $$\mathrm{Macroprecision}=\frac{1}{n}\sum_{i=1}^{n}\frac{TP_{i}}{TP_{i}+FP_{i}}$$

(9) $$\mathrm{Macrorecall}=\frac{1}{n}\sum_{i=1}^{n}\frac{TP_{i}}{TP_{i}+FN_{i}}$$

(10) $$\mathrm{Macro}\text{-}F1=\frac{2\times \mathrm{Macro}\text{-}\mathrm{Precision}\times \mathrm{Macro}\text{-}\mathrm{Recall}}{\mathrm{Macro}\text{-}\mathrm{Precison}+\mathrm{Macro}\text{-}\mathrm{Recall}}$$

(11) $$\mathrm{Accuracy}=\frac{TP+TN}{TP+FP+FN+TN}$$

where summation is after the identified classes, *i* denotes the class number, and *n* the number of considered classes. Table 5 shows the values of the statistical measures for the mode shape identification examples discussed earlier in the paper.

The proposed method for mode shapes identification gives precise results; its testing accuracy reaching 100% (see Figure 5 and Table 5) means that errors occur rarely enough to consider the proposed approach as error-free. The verification of mode shapes identification, performed on models with different materials (see Figure 6), confirms the accuracy and robustness of the method. This seems to indicate that the application of this method in SHM tasks and numerical models updating can significantly improve their accuracy and reliability.

The network CNN-O can correctly identify mode shapes of the model with varying number of composite layers and different materials. The sensitivity of the proposed method to the variation of other parameters, such as selected topological parameters like shell thickness, should be now verified.

The network CNN-N handles noised input data well (see Figure 7; the testing accuracy of mode shapes identification—even for the highest considered level of noise—is still close to 100%. This feature is extremely important from the point of view of the application of the method for identification of mode shapes obtained from experimental measurements; the method is based on a highly reduced description of the identified mode shape (only 1/242 of FEM-generated mode shape data is taken into consideration during the identification process).

The proposed method—namely CNN-D—can also be used in cases where the analyzed structure is exposed to local material degradation (caused, e.g., by corrosion or delamination). The material constants degradation localized in a selected zone affects the identification accuracy (see Figure 9a). However, the accuracy is still high and can be further improved by the application of the two-stage network learning, where the first stage of learning only uses as learning patterns the mode shapes obtained from the model without the local material degradation, and in the second stage of learning the patterns obtained from the model with the local material degradation are taken into account (see Figure 10). The obtained accuracy of mode shape identification exceeds 98% (see Figure 9b) when the network task is to assess only the class of mode shape, and 90% (see Table 4) in the case of a simultaneous identification involving also the state of the model (with or without local material degradation).

Finally, the method allows not only to identify the mode shapes; based on the identification results, it is possible to identify the state of the whole model. Depending on the approach applied (PA or CA), the accuracy of identification of the occurrence of local degradation of the model is close to 100% (CA approach; see Figure 12b) or even almost perfect (PA approach; see Figure 13b). Verification performed on the model with a different material than considered during network learning (see Figure 13c) proves again that the procedure is not sensitive to material change. Moreover, the results are precise even when the input data is significantly reduced: the reduction of the number of considered first mode shapes from 22 to 8 does not cause a significant decrease of the identification accuracy (see Figure 14), the procedure can still identify the appearance of even very small areas with material degradation (see Figure 15b).

It has to be firmly stated that the knowledge of the natural frequencies is not needed to identify a state of the model (with or without local material degradation). Moreover, the model studied, unlike many examples available in the relevant literature, is a three-dimensional model; therefore, the application of shape descriptors widely applied in the analysis of two-dimensional cases is not directly possible.

The main drawback of the proposed procedure is that it requires an analytical procedure for identifying the vibration mode shapes when preparing the patterns for network learning. However, the learning patterns can be, as shown above, only prepared for an intact model so that the analytical procedure must be fine-tuned to this particular case. This limitation should be taken into account when using the proposed approach.

## 6. Conclusions

The aim of the paper was to discuss a newly developed approach for the identification of vibration mode shapes of multilayer composite structures. The procedure based on convolutional networks has proved its effectiveness and robustness to noisy input data and can be thus applied applied in optimization and SHM problems.

The proposed method can successfully identify mode shapes of the composite cylinder; the change of the material or even occurrence of local material degradation do not affect the accuracy of the method. Moreover, the application of the proposed identification method allows identifying the material degradation occurrence.

Further research should be carried out; among others, the following problems should be addressed:

- application of graphical images to represent mode shapes rather than numerical data,
- application of the proposed method of mode shape identification in optimization tasks,
- identification of the location of the area of local material degradation.

## Figures and Tables

**Table 1:** The applied convolutional neural network (CNN) architecture (25 classes case).

Layer	Input	Kernel	Kernel	Dimension	Activation
Number	Type	Number	Size	of Data	Function
1	Convolution	33	{2,5}	33 × 2 × 16	
2	Batch normalization			33 × 2 × 16	
3	Activation			33 × 2 × 16	ReLU
4	Convolution	66	{2,5}	66 × 1 × 12	
5	Batch normalization			66 × 1 × 12	
6	Activation			66 × 1 × 12	ReLU
7	Convolution	33	{1,3}	33 × 1 × 10	
8	Batch normalization			33 × 1 × 10	
9	Activation			33 × 1 × 10	ReLU
10	Pooling		{1,2}	33 × 1 × 5	
11	Flatten			165	
12	MLP			75	
13	MLP			25	
14	SoftMax			25	Softmax
	Output “class”				

**Table 2:** Number of wrong CNN identification cases (among all considered cases).

	Learning	Testing
CNN-O mode shapes identification (Figure 5)	7 (96,000)	3 (96,000)
Testing for a different material (Figure 6)	—	0 (19,200)
Noised mode shapes, $\sigma_{1}=0.00005$ and $\sigma_{2}=0.05$	8 (96,000)	6 (96,000)
Noised mode shapes, $\sigma_{1}=0.0001$ and $\sigma_{2}=0.10$	9 (96,000)	16 (96,000)
Noised mode shapes, $\sigma_{1}=0.0005$ and $\sigma_{2}=0.25$ (Figure 7)	12 (96,000)	20 (96,000)

**Table 3:** Number of wrong feedforward neural network (FFNN) identification cases (among all considered cases).

	Learning	Testing
FFNN mode shapes identification	62 (96,000)	17 (96,000)
Noised mode shapes, $\sigma_{1}=0.0005$ and $\sigma_{2}=0.25$	164 (96,000)	162 (96,000)

**Table 4:** The results of identification of mode shapes with and without local material degradation.

	Learning		Testing	
	*n* = 4	*n* = 16	*n* = 8	*n* = 32
24 classes				
Patterns with no material degradation	95.4%	98.2%	94.3%	96.8%
Resultant accuracy	96.8%		95.6%	
Patterns with material degradation	80.9%	84.7%	75.6%	80.0%
Resultant accuracy	82.8%		77.8%	
Overall accuracy, with and without material degradation	91.6%		89.0%	
25 classes				
Patterns with no material degradation	95.5%	97.2%	93.0%	97.1%
Resultant accuracy	96.4%		95.1%	
Patterns with material degradation	87.9%	89.2%	80.1%	80.7%
Resultant accuracy	88.6%		80.4%	
Overall accuracy, with and without material degradation	93.3%		89.7%	
5 classes, two stage learning; CNN-D				
Patterns with no material degradation	97.2%	98.2%	93.8%	95.9%
Resultant accuracy	97.7%		94.9%	
Patterns with material degradation	92.2%	93.3%	81.4%	85.8%
Resultant accuracy	92.8%		83.6%	
Overall accuracy, with and without material degradation	95.9%		90.7%	

**Table 5:** Statistical measures describing the accuracy of vibration mode shapes identification.

	Models without Material Degradation					With Degradation	
	CNN-O		CNN-O	CNN-N		CNN-O	CNN-D
	Learn	Test	New Material	Learn	Test	Figure 9a	Figure 9b
Macro-Precision	0.9999	0.9999	1.0000	0.9998	0.9997	0.8453	0.8985
Macro-Recall	0.9999	0.9998	1.0000	0.9996	0.9998	0.8006	0.9149
Macro-F1	0.9997	0.9998	1.0000	0.9997	0.9997	0.8223	0.9066
Accuracy	0.9999	1.0000	1.0000	0.9996	0.9998	0.9601	0.9811

## Data Availability

The data underlying this article will be shared on reasonable request from the corresponding author.

## Abbreviations

The following abbreviations are used in this manuscript:

CNN	Convolutional neural network
FFNN	Feedforward neural network
FE	Finite element
FEM	Finite element method
PA	Probability approach
CA	Counting approach
TP	True positives
TN	True negatives
FP	False positives
FN	False negatives
